# Whole-exome analysis reveals novel somatic genomic alterations associated with cell of origin in diffuse large B-cell lymphoma

**DOI:** 10.1038/bcj.2017.33

**Published:** 2017-04-21

**Authors:** B A Manso, K Wenzl, Y W Asmann, M J Maurer, M Manske, Z-Z Yang, S L Slager, G S Nowakowski, S M Ansell, T E Witzig, A L Feldman, L Rimsza, B Link, J R Cerhan, A J Novak

**Affiliations:** 1 Department of Immunology, Mayo Clinic, Rochester, MN, USA; 2 Division of Hematology, Mayo Clinic, Rochester, MN, USA; 3 Division of Biomedical Statistics and Informatics, Mayo Clinic, Rochester, MN, USA; 4 Department of Laboratory Medicine and Pathology, Mayo Clinic, Rochester, MN, USA; 5 Department of Laboratory Medicine and Pathology, Mayo Clinic, Scottsdale, AZ, USA; 6 Department of Hematology, Oncology and Blood and Marrow Transplantation, University of Iowa, Iowa City, IA, USA; 7 Division of Epidemiology, Mayo Clinic, Rochester, MN, USA

Diffuse large B-cell lymphoma (DLBCL) is the most common lymphoma, with an estimated 27 650 cases diagnosed in 2016.^1^ Gene expression profiling has identified three subtypes of DLBCL; germinal center B-Cell like (GCB), activated B-cell like (ABC) and primary mediastinal B-cell lymphoma (PMBL).^2^ This molecular heterogeneity is indicative of a unique cell of origin (COO) giving rise to each subtype and is associated with clinical outcome, with treatment, leading to remission in ~80% of GCB patients but only ~50% in ABC patients.^3^ The poor prognosis of ABC-DLBCL is distinguished by constitutive activity of the NF-κB pathway,^4^ which has also been associated with drug resistance in all subtypes of DLBCL.^5^ However, heterogeneity remains an issue within COO subtypes and there is a need for further classification in the current era of COO-specific therapeutic targeting. Sequencing of DLBCL tumors has identified a panel of mutations that associate with COO, however, much of this work was performed using limited gene panels or traditional Sanger sequencing methods across multiple patient cohorts. Furthermore, most previous studies defined COO using the Hans method, which only has ~80% concordance with gene expression profiling.^6^ A comprehensive genomic analysis of COO, including both mutations and copy number analysis, on a clinically defined set of DLBCL cases with accurate COO has not been performed.

Discovery of genetic biomarkers that provide insight on tumor biology, are predictive of treatment outcome, and identify therapeutic targets are central to the future of precision therapy in DLBCL. In an effort to use genomics as a clinical predictor of therapeutic response, we used the whole-exome sequencing (WES) data from 51 DLBCL tumors to identify novel somatic genomic alterations associated with outcome in immunochemotherapy-treated DLBCL patients.^7^ Building on this effort, we next utilized the WES data on 58 DLBCL patients with COO and performed a comprehensive genetic analysis to better define the genomic differences between GCB and ABC DLBCL. Cell of origin was determined using the gene expression profile (GEP) data (*n*=37)^8^ or NanoString technology (*n*=21).^9^ All available cases (*n*=44) were screened for a *MYC, BCL2* and *BCL6* rearrangement by FISH as previously described.^7^ WES of DNA from 58 newly diagnosed frozen DLBCL tumors and paired germline DNA was performed at the Broad Institute and somatic mutations and exon-level copy number alterations (CNAs) were called as previously described.^7, 10^ A CNA was called for each chromosomal region based on the loss or gain of the following genes: 10q11.21-10q24.23 loss (*PTEN*), 4q12-4q35.2 loss (*IGJ*), 7q11.1-7q36.3 gain (*CDK14*), 3q12.1-3q29 loss (*TP63*), 2p13-2p12 gain (*REL*), 6q21 loss (*PRDM1*), 9p21 loss (*CDKN2B*), 18q21.33 gain (*BCL2*) and 9p24.1 gain (*CD274*). We estimated measures of association using odds ratios and report the association of genomic variants with COO using a *χ*^2^ test. For this exploratory study, we used a nominal level of statistical significance (*P*<0.05), and we did not adjust for multiple testing. While all mutations and CNA identified in the 58 cases were analyzed for their association with COO, the variants reported in this study include (1) all mutations and CNA that had an association with COO (*P*<0.05), and (2) those previously identified as drivers of DLBCL.^10, 11, 12, 13, 14^ Patient characteristics are described in Table 1.

A total of 27 patients were classified as GCB, 26 as ABC, and five were unclassified (Figure 1a). In total, 37 genomic abnormalities are reported for their association with either GCB (Figure 1a, red boxes) or ABC (green boxes), or neither (blue boxes). We find that mutations in *BCL2*, *TNFRSF14*, *GNA13* and *FAT3* significantly associate (*P*<0.05) with the GCB subtype, largely agreeing with previous reports.^15, 16^ Mutations in *P2RY8*, *EZH2* and *FOXO1* have also been reported as GCB driver mutations and we find that mutations in these genes are restricted to GCB. *MYC* double-hits (MYC-DH, gene rearrangements of *MYC* with *BCL2* and/or *BCL6*) were present only in GCB patients. Mutations in *MYD88* associated (*P*=0.03) with ABC in our dataset, while mutations in *CD79B* and *TNFAIP3*, both known to associate with ABC,^17, 16^ trend towards association. The remaining mutations reported did not strongly associate with either ABC or GCB, suggesting that there is common biology underlying both subgroups.

In addition to mutational patterns, we identified several CNAs across both groups. Chromosomal losses (*P*<0.05) associated with GCB DLBCL were found at chromosomes 10q11.21-10q24.23 and 4q12-4q35.2, with a gain at 7q11.1-1q36.3 trending towards GCB (*P*=0.07). No copy-number variation was observed to directly associate with ABC patients however, a loss at 9p21 and gains at 18q21.33 trended with the ABC subtype, supporting previous reports.^18^ A gain in 2p13-2p12 has been reported as being specific for GCB,^18^ but our data identify it occurring in both subtypes. Additionally, losses at 3q12.1-3q29 and 6q21 occurred in both subtypes.

In an effort to further understand genomic differences between DLBCL subtypes, we evaluated the relative percentage of each genomic instability (Figure 1b). Of the reported genomic alterations, 7/37 (18.9%) were only observed in GCB patients, whereas 2 out of 37 (5.4%) were specific to the ABC subtype. The majority (28 out of 37, 75.7%) overlapped between ABC and GCB, potentially indicative of similar biology between subtypes.

Patients diagnosed with ABC DLBCL have been reported to have a worse clinical prognosis than GCB patients,^15, 18^ likely due to chronic B-cell receptor signaling and constitutive activation of NF-κB from acquired mutations in upstream genes such as *CD79B*, *CARD11* and *MYD88*. Interestingly, we identified seven patients (Figure 1a, No. 47–53) within our ABC cluster that do not exhibit any of the 37 genomic alterations reported here that require further genomic study to better resolve the predictive survival analysis of DLBCL patients. Four additional cases (Figure 1a, No. 43–46) had only one genomic alteration. This may be indicative of other, yet to be identified genomic instabilities that may contribute to poor clinical outcome in these cases. Taken together, this analysis has further characterized the genetic profile of each COO subtype and has identified novel GCB CNAs that may contain candidate genes that provide insight on tumor biology and offer potential targets for therapy. Collectively, these data provide insight on the genetic heterogeneity of DLBCL and identify genomic variants that can inform subtype-specific therapy.

## Figures and Tables

**Figure 1:**
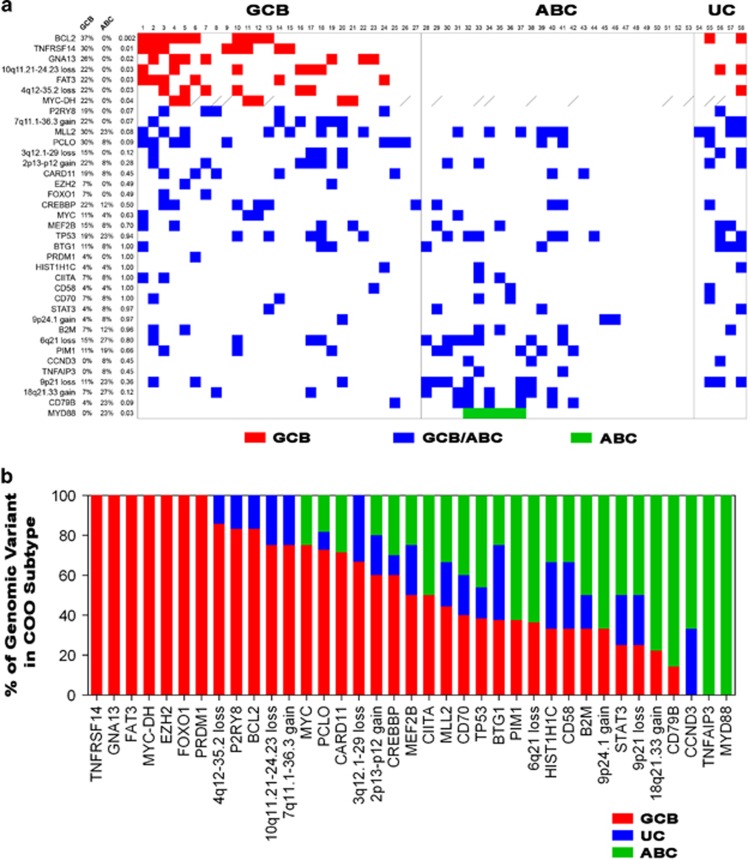
Observed genomic alterations in 58 DLBCL patients. (**a**) 58 DLBCL patients were stratified into either GCB, ABC or unclassified (UC) DLBCL subtypes. Known and statistically significant genomic alterations were identified and sorted by associated significance within each subtype and by those having no direct association. The percent prevalence and statistical significance of each genomic variant within each subtype is reported. Red and green squares indicate genomic alterations statistically associated (*P*⩽0.05) with GCB or ABC, respectively. Blue squares represent nonsignificant genomic alterations. Hashed boxes represent no data available. (**b**) Stratification of genomic alteration identified within COO subgroups, when present. Red, green and blue bars represent the respective GCB, ABC and UC frequency within each genomic instability, whereas blue bars represent the contribution from unclassified cases.

**Table 1:** DLBCL patient characteristics

*Characteristic*	*ABC (*N=*26)*	*GCB (*N=*27)*	*Unclassified (*N=*5)*	P*-value*
Diagnosis age, median (range); IQR	60 (28–84); 55–71	66 (26–88); 60–75	68 (62–77); 66–70	0.2061
Age >60	13 (50.0%)	20 (74.1%)	5 (100.0%)	0.0707
Male	17 (65.4%)	15 (55.6%)	3 (60.0%)	0.4646
PS 2+	3 (11.5%)	6 (22.2%)	0 (0.0%)	0.3004
Ann arbor stage III–IV	23 (88.5%)	16 (59.3%)	2 (40.0%)	0.0159
2+ extranodal sites group	6 (23.1%)	3 (11.1%)	1 (20.0%)	0.2461
LDH >ULN	17 (65.4%)	16 (61.5%)	3 (60.0%)	0.7734
				
*IPI*				
0–1	4 (15.4%)	8 (29.6%)	2 (40.0%)	0.4829
2	10 (38.5%)	6 (22.2%)	1 (20.0%)	
3	9 (34.6%)	9 (33.3%)	1 (20.0%)	
4 or 5	3 (11.5%)	4 (14.8%)	1 (20.0%)	
B symptoms	7 (26.9%)	8 (29.6%)	1 (20.0%)	0.8269
Bulky disease	2 (7.7%)	5 (18.5%)	1 (20.0%)	0.2445

Abbreviations: ABC, activated B-Cell like; GCB, germinal center B-cell like
